# Ninjin’yoeito suppressed the onset of arthritis, pain, and muscle atrophy in rheumatoid arthritis model mice

**DOI:** 10.3389/fphar.2022.974380

**Published:** 2022-12-19

**Authors:** Takeshi Suginohara, Megumi Kawaguchi, Seiwa Michihara, Nina Fujita, Li-Kun Han, Ryuji Takahashi

**Affiliations:** Kampo Research Laboratories, Kracie Pharma Ltd., Toyama, Japan

**Keywords:** Ninjin’yoeito, rheumatoid arthritis, collagen-induced arthritis (CIA) model, sarcopenia, muscle atrophy

## Abstract

Rheumatoid arthritis is one of the most common diseases in orthopedic surgery. The main symptoms are joint pain and systemic symptoms. In recent years, rheumatoid arthritis is known to cause sarcopenia. Ninjin’yoeito (NYT), a traditional Japanese medicine, has been prescribed for patients with post-illness or post-operative weakness, fatigue, loss of appetite, rash, cold limbs, and anemia. In addition to its traditional use, NYT has been prescribed for treating frailty in gastrointestinal, respiratory, and urinary functions. Further, NYT is known to be effective in suppressing muscle atrophy in the prior literature. The present study aimed to investigate whether NYT suppresses various symptoms of the Collagen-induced arthritis (CIA) model. Long-term administration of NYT inhibited the increases in arthritis scores, decreases pain threshold, and muscle atrophy in the CIA model. In addition, NYT inhibited the elevation of the plasma IL-6 level. These results suggest that NYT may have therapeutic effects on symptoms, muscle atrophy and increase in plasma IL-6 level caused by rheumatoid arthritis.

## Graphical Abstract

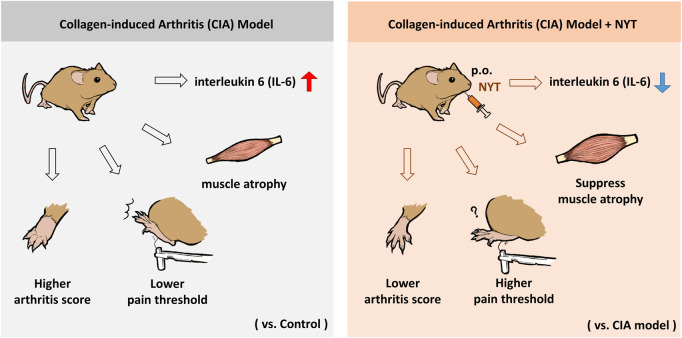

## 1 Introduction

Rheumatoid arthritis is an autoimmune disease that causes inflammation of the joints. The incidence rate of rheumatoid arthritis is currently estimated to be 0.4%–0.5% in the Japanese population (Chugai Pharmaceutical, 2020). Approximately 1% of the population over the age of 30 is thought to be affected, indicating that the susceptible age group is individuals aged 30–50 years. Characteristic symptoms include swelling of the joints of the hands and feet and intense pain, even during rest. Systemic symptoms include fever, fatigue, and anorexia, and in some cases, inflammation spreads throughout the body to the lungs, blood vessels, and other organs. As the pathophysiological process progresses, the cartilage and the bones break down until the joints lose functionality and eventually become deformed. In most cases of rheumatoid arthritis, spontaneous remission does not occur, leading many patients to seek treatment by an orthopedic surgeon. In recent years, there have been reports of clinical cases of sarcopenia comorbidity in addition to the systemic disorders previously associated with rheumatoid arthritis, and thus sarcopenia is now viewed as an additional problem (Hanh-Hung et al., 2011; Matsumoto et al., 2015).

Sarcopenia refers to the decreased skeletal muscle mass and strength associated with aging. Sarcopenia is viewed as an underlying disease associated with frailty, which is an important issue to consider for extending the healthy life expectancy in a super-aging society. When sarcopenia occurs, it not only affects the basic activities and behaviors of daily life, but it also causes other diseases to become increasingly severe and impacts survival duration (The Japan Foundation for Aging and Health, Healthy Aging Network., 2021). There are two types of sarcopenia. Primary sarcopenia is mainly aging-related, while secondary sarcopenia is caused by decreased activity and worsening disease and nutritional status in addition to aging. In the present study, we focused on secondary sarcopenia occurring as a result of rheumatoid arthritis.

*Ninjin’yoeito* (NYT) is a *kampo* (traditional remedy) that consists of 12 types of crude drugs, including ginseng (Table 1). Clinical trials have reported that NYT is effective on several conditions related to aging, such as decreased muscle strength, deterioration of muscle quality, and muscle atrophy (Sakisaka et al., 2017; Naoya et al., 2018). We have previously reported that NYT exerted an anti-muscle atrophy action in a variety of experimental animal models (Takahashi et al., 2018; Takemoto et al., 2021). It has also been reported that NYT was beneficial in an autoimmune disease model (Nakai et al., 1993). Therefore, we believe that it is highly likely that NYT may similarly be effective against rheumatoid arthritis, which is an autoimmune disease, as well as rheumatoid arthritis-induced sarcopenia.

**TABLE 1:** Composition (in a daily dose ^a^) of *Kampo* Formula *Ninjin’yoeito* (NYT).

Ingredients			Amount (g)
English name	Latin name	Original plant source and medicinal part	
Poria Sclerotium	Poria	The sclerotium of Wolfiporia cocos Ryvarden et Gilbertson (Poria cocos Wolf)	4
Japanese Angelica Root	Angelicae Acutilobae Radix	The root of Angelica acutiloba Kitagawa or Angelica acutiloba Kitagawa var. Sugiyamae Hikino	4
Rehmannia Root	Rehmanniae Radix	The root of Rehmannia glutinosa Liboschitz var. purpurea Makino or Rehmannia glutinosa Liboschitz	4
Atractylodes Rhizome	Atractylodis Rhizoma	The rhizome of Atractylodes japonica Koidzumi ex Kitamura or Atractylodes macrocephala Koidzumi (Atractylodes ovata De Candolle)	4
Ginseng	Ginseng radix	The root of Panax ginseng C. A. Meyer (Panax schinseng Nees)	3
Cinnamon Bark	Cinnamomi cortex	The bark of the trunk of Cinnamomum cassia J. Presl	2.5
Citrus Unshiu Peel	Citri Unshiu Pericarpium	The pericarp of the ripe fruit of Citrus unshiu Markowicz or Citrus reticulata Blanco	2
Polygala Root	Polygalae Radix	The root or root bark of Polygala tenuifolia Willdenow	2
Peony Root	Paeoniae Radix	The root of Paeonia lactiflora Pallas	2
*Astragalus* Root	Astragali Radix	The root of *Astragalus* membranaceus Bunge or *Astragalus* mongholicus Bunge	1.5
Schisandra Fruit	Schisandrae Fructus	The fruit of Schisandra chinensis Baillon	1
*Glycyrrhiza*	Glycyrrhizae Radix	The roots and stolons of *Glycyrrhiza* uralensis Fischer or *Glycyrrhiza* glabra Linné	1

^a^ Approximate 6,700 mg of water-dried extract of NYT, was prepared in the GMP-standardized factory of Kracie Pharmaceutical, Ltd. (Qingdao, China) with the composition described above.

Thus, we created a rheumatoid arthritis model induced by intradermal administration of collagen and investigated the beneficial action of NYT on the symptoms of rheumatoid arthritis and rheumatoid arthritis-associated sarcopenia.

## 2 Materials and methods

### 2.1 Medicines

*Ninjin’yoeito* extract (Lot.: E1510311A0) was manufactured by the GMP Pharmaceutical Factory of Kracie Pharma, Ltd. (Qingdao, China). The quality of each plant material was identified by external morphology and authenticated by marker compounds of the plant specimens (e.g., glycyrrhizic acid, paeoniflorin, hesperidin, etc.) according to the Japanese Pharmacopoeia and standards of Kracie Pharma, Ltd. The analytical method and 3D-High Performance Liquid Chromatography (HPLC) profile of the NYT extract were the same as those reported previously (Murata et al., 2018).

### 2.2 Animals

The seven-week-old male DBA/1 J mice used in our experiments were purchased from Japan SLC, Inc. (Shizuoka, Japan). Three or four animals each were kept in cages with floors under the following conditions: Room temperature 23 ± 2°C; humidity 55 ± 10%; 12-h light/dark cycle (light on at 8:00, and light off at 20:00 light). During the study period, they animals had free access to food and water.

This animal study was reviewed and approved by the Experimental Animal Care Committee of Kracie Pharma, Ltd. It was conducted in accordance with the Kracie Pharma Co., Ltd. Kampo Laboratory Guidelines for Animal Experiments and the Standards Relating to the Care and Management of Experimental Animals (Notification No. 6, 27 March 1980 of the Prime Minister’s Office, Japan).

### 2.3 Collagen-induced arthritis (CIA) model

In this study, we prepared the Collagen-induced Arthritis (CIA) model, which is the most used animal model of RA (Trentham et al., 1977). Based on their body weight at the time the seven-week-old male DBA/1 J mice were delivered, we divided the animals into four groups: a No-Treatment group, a Control group, a 300 mg/kg NYT (NYT300) group, and a 1,000 mg/kg NYT (NYT1000) group (*n* = 7 for all). After a 1-week preliminary period, the Control group, NYT300 group, and NYT1000 group were given intradermal administration of an emulsion made of collagen (Collagen Research Center, type II collagen powder, K42, Tokyo, Japan) and an adjuvant under inhalation of the anesthetic Isoflurane. The emulsion was administered at a dose of 0.1 ml in the animals’ tails (at a site approximately 5 mm from the base of the tail). This treatment was performed twice. The first time, we utilized an emulsion made with a complete adjuvant (Nacalai, adjuvant complete, 263,810, Kyoto, Japan). The second time, we performed intradermal administration of an emulsion made of an incomplete adjuvant (Nacalai, adjuvant incomplete, 263910, Kyoto, Japan) 3 weeks after the first administration. The date of the second antigen administration was designated as the experimental day 0 and used as such for all subsequent records.

### 2.4 Drug treatment

At 8 weeks after the second antigen administration, the test drug was administered. Oral administration of the NYT extract solution was performed once per day for a 6-week period at a dose of 0.1 ml per 10 g of body weight so that the dose was 300 and 1,000 mg/kg, respectively in the NYT300 and NYT1000 groups. The No-Treatment and Control groups were given orally-administered distilled water in the same dose of 0.1 ml per 10 g of body weight.

### 2.5 Arthritis score

The arthritis score was measured using the Chondrex Inc. Arthritis Scoring Method (Nakai, S., 1993). Following the second antigen administration, measurements were taken every other day. Assessments were performed on three joints (finger, instep and base of toe, and wrist) with a possible maximum score of 4 points per finger or toe (possible maximal score per animal: 16 points).

### 2.6 Pain test

Mechanical allodynia in mice was evaluated using the von Frey test. In the von Frey test, a series of calibrated von Frey filaments (Touch-Test Sensory Evaluator, North Coast Medical, Inc., Morgan Hill, CA) with a bending force 0.008 g, 0.02 g, 0.04 g, 0.07 g, 0.16 g and 0.4 g were applied to the midplantar skin of each hind paw at a rate of once per second. Specifically, the paw was stimulated 10 times at a speed of once per second in the order from the filament with the lowest strength and when escape behavior was noted once or more, the mouse was judged as positive. Then, stimuli were added using the one-step weaker filament when the animal was positive and using the one-step stronger filament when the animal was negative. Stimulation was repeated until observing positive and negative responses to stimulation with two continuous filament types, respectively, and the filament strength to which a positive response was observed was recorded as the pain threshold. Measurements were performed 1 week and 4 weeks after the second antigen administration. The mice were transferred one at a time to a steel mesh cage with a glass container and allowed to acclimatize for a period of approximately 1 h. To reduce measurement variability, the glass container was placed on top of the mice to restrict their movements during measurement. This experiment was conducted as a blind test.

### 2.7 Necropsy

On the final day of the experiment, the mice underwent necropsy under anesthetic Isoflurane inhalation. Three types of muscles were excised, namely the soleus muscle, the plantaris muscle, and the gastrocnemius muscle, and their wet weights were measured.

### 2.8 Plasma analysis

We once again purchased seven-week-old male DBA/1J mice and conducted the same group allotments. We created the same CIA model as described above and administered NYT for a period of 4 weeks. NYT extract (Lot.: E1510311A0) solution was administered orally at a dose of 0.1 ml per 10 g of body weight once per day for a period of 6 weeks so that 300 and 1,000 mg/kg, respectively were administered to the NYT300 group and the NYT1000 group. The Non-Treat and Control groups were given oral administration of distilled water according to the same parameters. At week four of the experiment, blood samples were taken using syringes treated with heparin and with the animals under Isoflurane inhalation anesthetic. The samples were centrifuged for 10 min at 3,000 rpm and at a temperature of 4°C. The centrifuged plasma was then placed into frozen storage at −40°C. We then had GeneticLab Co., Ltd. measure interleukin 6 (IL-6) and tumor necrosis factor–α (TNF-α), which are types of inflammatory cytokines.

### 2.9 Statistical analysis

Statistical analysis was performed on the arthritis score, pain threshold, muscle wet weight, and cytokine level in blood using EZR (Saitama Medical Center, Jichi Medical University, Saitama, Japan; Kanda, 2013). In cases in which one-way analysis of variance (ANOVA) or the Kruskal-Wallis test of the No-Treatment, Control, NYT300, and NYT1000 groups showed differences, Dunnett’s test or the Steel test was used to perform multiple comparisons. Analysis of the muscle wet weight was conducted using the parametric statistics. Analyses of the arthritis score, pain threshold, and blood cytokine level were conducted using non-parametric statistics. The correlation between the pain threshold and arthritis score was determined using Spearman’s rank correlation coefficient.

## 3 Results

### 3.1 Effect of NYT on arthritis score

**FIGURE 1:**
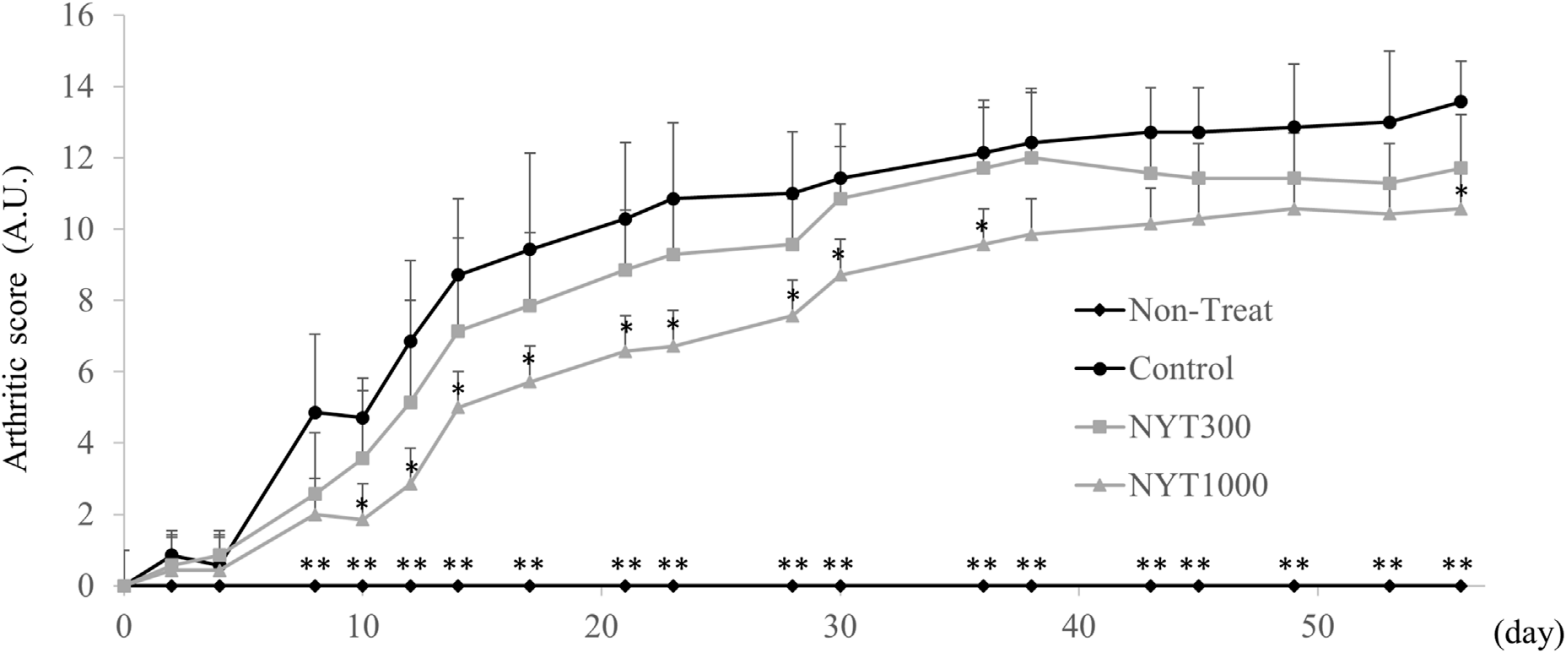
Effect of *Ninjin’yoeito* (NYT) on arthritic score in collagen-induced arthritis (CIA) mice (*n* = 7). Values are expressed as means ± S.D. ∗*p* < 0.05, ∗∗*p* < 0.01 vs control group by steel test.

The arthritis score of the Control group became higher than that of the No-Treatment group starting on the eighth experimental day (Figure 1). We confirmed that, following administration of NYT, the arthritis score elevations in the CIA model were suppressed in the NYT1000 group starting on the 10th experimental day. A statistically-significant suppression of the arthritis score elevation as was not observed in the NYT300 and Control groups.

### 3.2 Effects of NYT on CIA-induced mechanical allodynia

**FIGURE 2:**
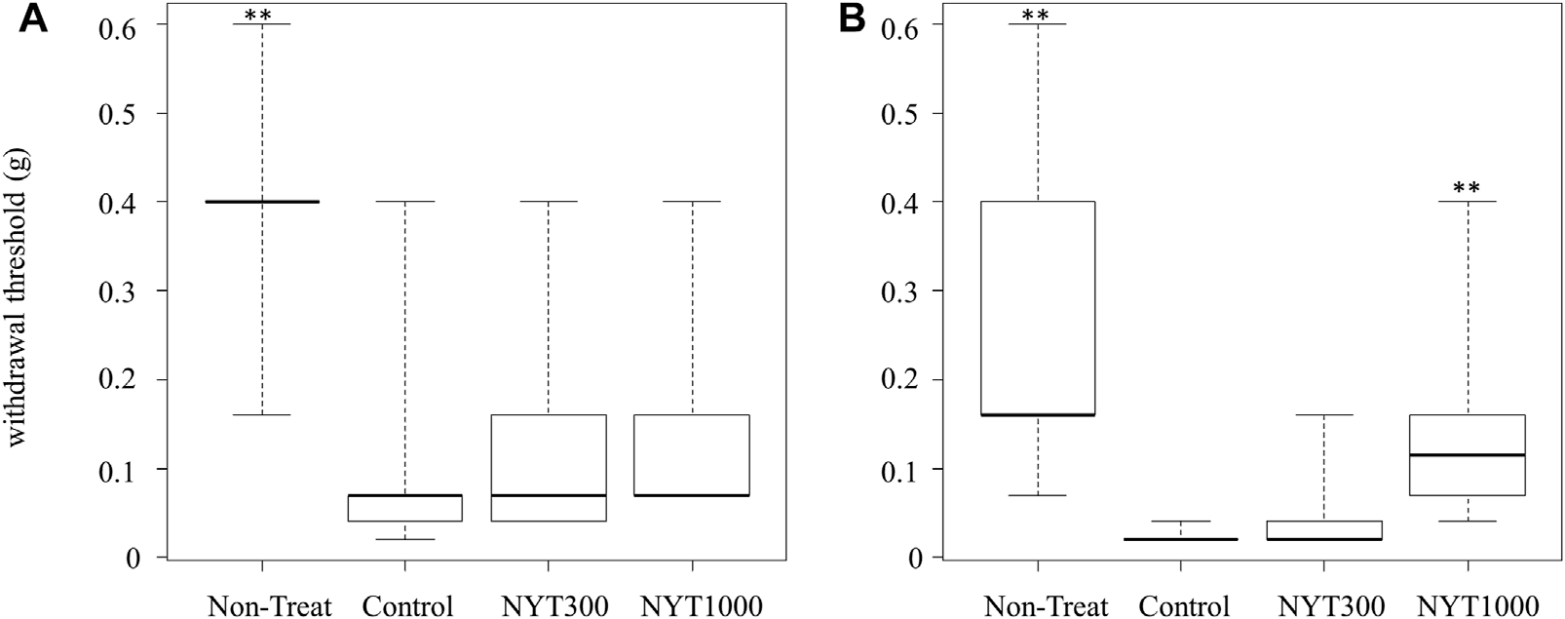
Effect of NYT on CIA-induced mechanical allodynia. **(A)** First week. **(B)** Fourth week after second antigen administration (Number of legs; each group *n* = 14). Values are expressed as median [25th percentile, 75th percentile]. ∗∗*p* < 0.01 vs. control group by steel test.

**FIGURE 3:**
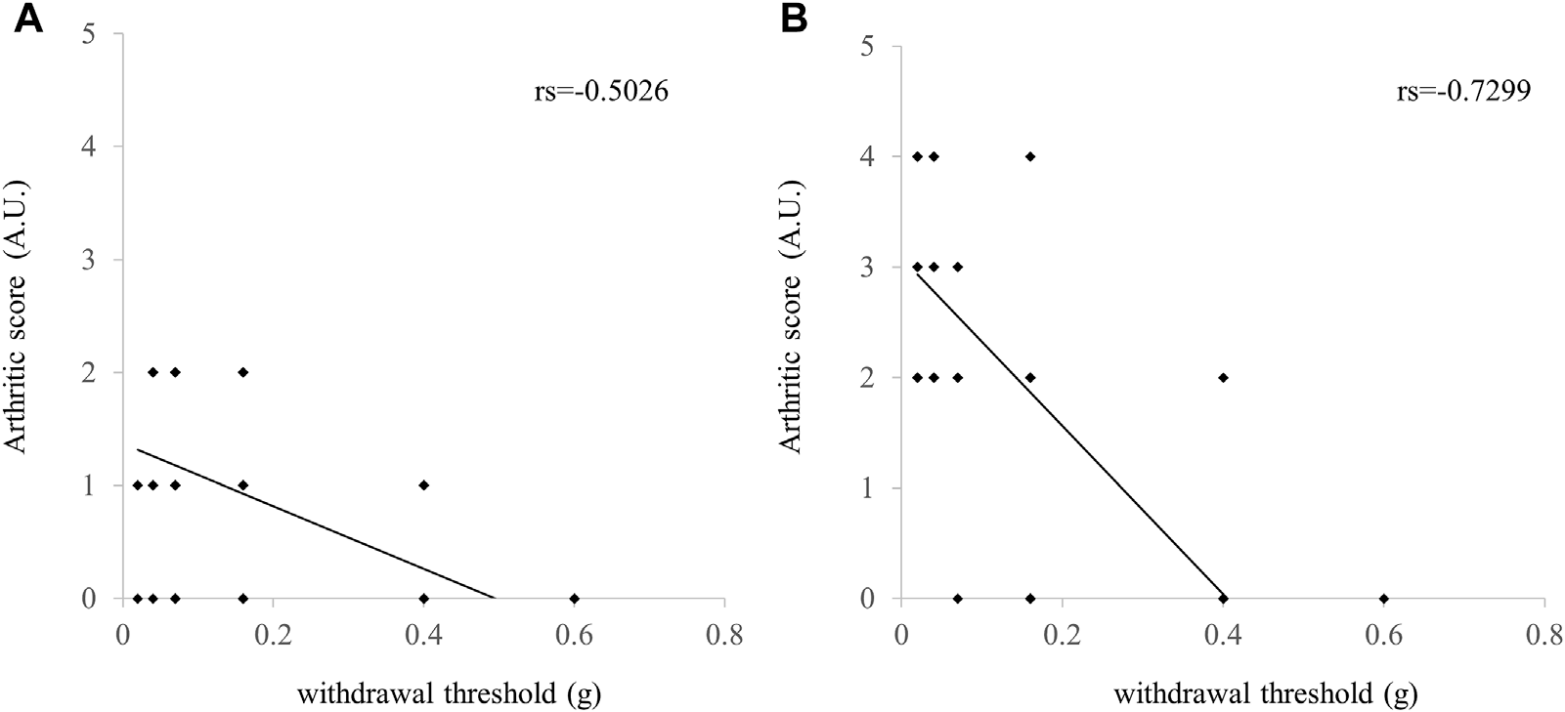
Correlation of arthritic score with mechanical allodynia. **(A)** First week. **(B)** Fourth week for the second antigen administration (Number of legs; total 56). Pearson’s correlation coefficient was used for the statistical analyses. *p* < 0.05 was considered statistically significant.

**FIGURE 4:**
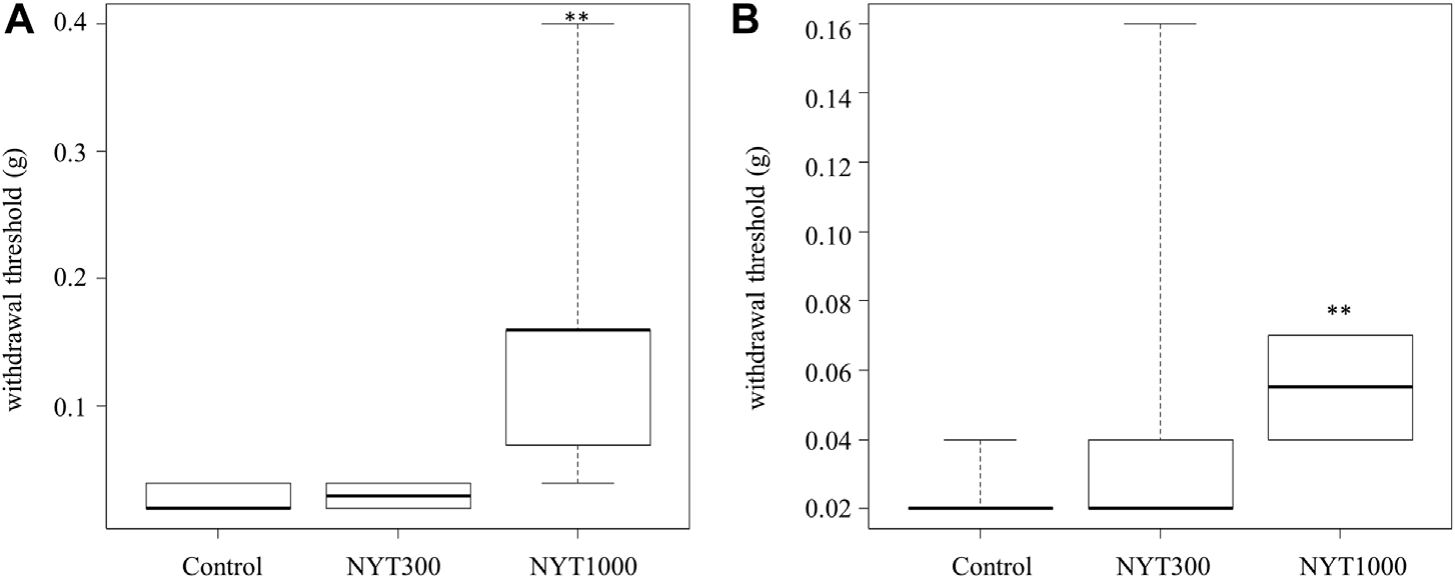
Effect of NYT on CIA-induced mechanical allodynia in same score group. **(A)** Group arthritic score 1 and 2 (Number of legs; Control *n* = 5, NYT300 *n* = 4, NYT1000 *n* = 10). **(B)** Group arthritic score 3 and 4 (Number of legs; Control *n* = 9, NYT300 *n* = 10, NYT1000 *n* = 4). Values are expressed as median Please clarify. ∗∗*p* < 0.01 vs. Control group by Steel test.

During the first experimental week, the pain threshold of the Control group became lower than that of the No-Treatment group (Figure 2A). NYT administration was not found to be effective against pain threshold elevation (Figure 2A). Similarly, during the fourth experimental week, it was found that the pain threshold of the Control group was lower than that of the No-Treatment group (Figure 2B). We confirmed that, following NYT administration, the decrease in the pain threshold of the CIA model was mitigated in the NYT1000 group. We did not observe significant effectiveness in mitigating the pain threshold decrease in the NYT300 group compared to the Control group. Our analysis of the correlation between the pain threshold and the arthritis score for the rear paw revealed a significant negative correlation (Figure 3). We also compared the pain thresholds of the Control, NYT300, and NYT1000 groups for the same arthritis score (score 1: 2 animals and score 3: 4 animals) during the fourth experimental week (Figure 4). The results of our analysis showed that for both scores, the NYT1000 group showed statistically significant elevations of the pain threshold compared to the Control group.

### 3.3 Effect of NYT on muscle wet weight

**FIGURE 5:**
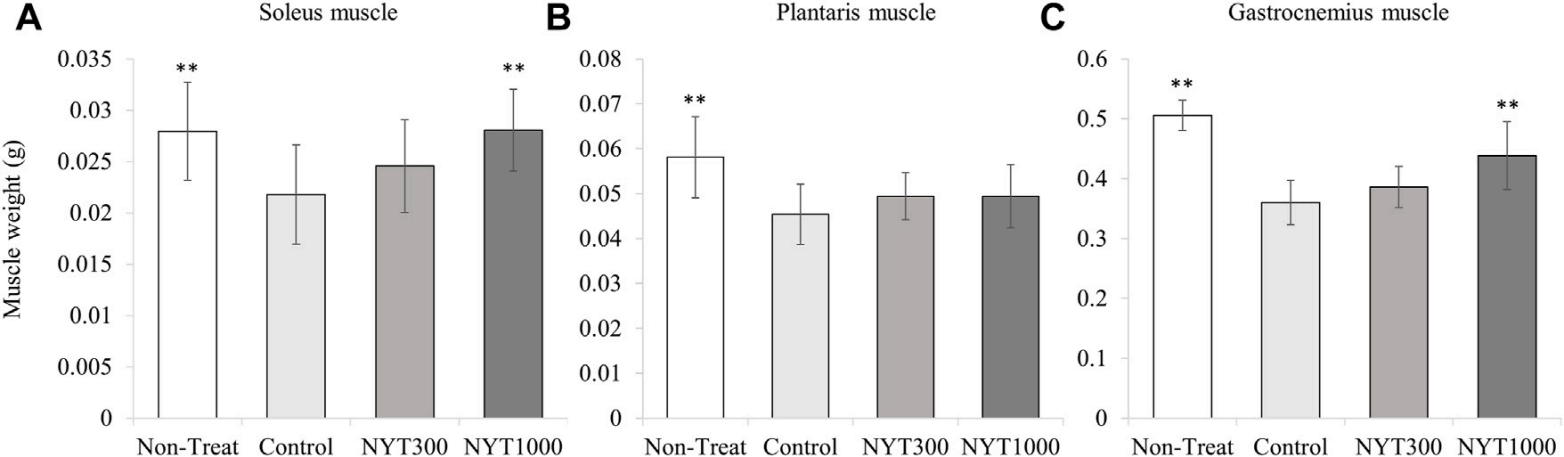
Effect of NYT on muscle weight. **(A)** Soleus muscle. **(B)** Plantaris muscle, **(C)** Gastrocnemius muscle (Number of legs; Each group *n* = 14). Values are expressed as means ± S.D. ∗∗*p* < 0.01 vs. control group by dunnett test.

**FIGURE 6:**
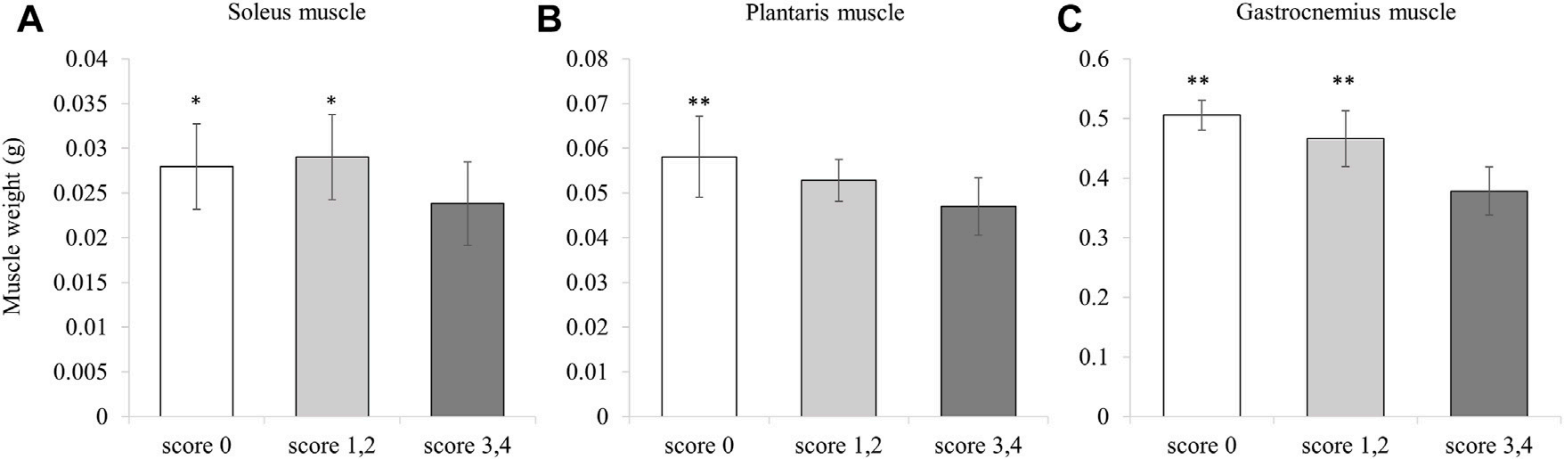
Effect of NYT on muscle weight in same score group. **(A)** Soleus muscle. **(B)** Plantaris muscle. **(C)** Gastrocnemius muscle (Number of legs; Score 0 *n* = 14, score 1,2 *n* = 8, score 3,4 *n* = 34). Values are expressed as means ± S.D. ∗*p* < 0.05, ∗∗*p* < 0.01 vs score 3,4 group by dunnett test.

**FIGURE 7:**
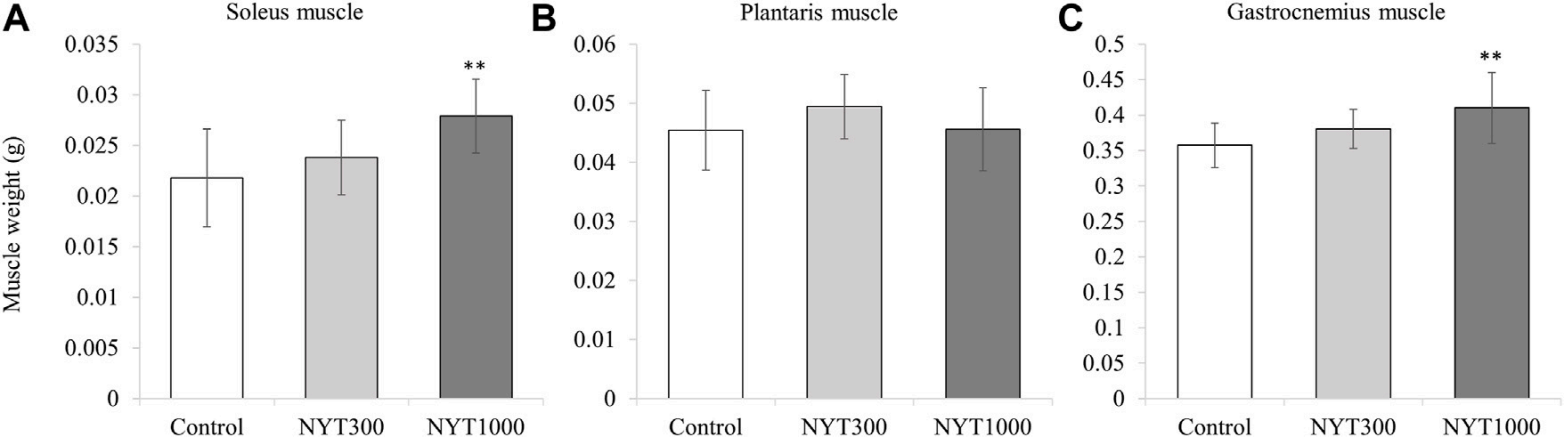
Effect of NYT on muscle weight in score 3,4 group. **(A)** Soleus muscle. **(B)** Plantaris muscle. **(C)** Gastrocnemius muscle (Number of legs; Control *n* = 14, NYT300 *n* = 13, NYT1000 *n* = 7). Values are expressed as means ± S.D. ∗∗*p* < 0.01 vs. control group by dunnett test.

We found the wet weights of all the skeletal muscles were significantly lower in the Control group than those in the No-Treatment group (Figure 5). For the soleus muscle and the gastrocnemius muscle, NYT administration was found to counteract muscle atrophy in the CIA model (Figures 5A, C). In contrast, there was no significant reversal of muscle atrophy for the plantaris muscle by NYT compared to the Control group (Figure 5B). We also compared the wet weight of the three types of skeletal muscle when divided based on the foot arthritis score (score 0, score 1 and 2, and score 3 and 4; Figure 6). In all skeletal muscles, the muscle wet weight decreased as the arthritis score increased. As an exploratory study, we conducted a comparative analysis of the animals with severe conditions (foot arthritis score 3 and 4) in the Control and NYT groups (Figure 7). The results showed that, in the case of the soleus and gastrocnemius muscles, the NYT1000 group showed significant reversal of muscle atrophy compared to animals in the Control group with the same arthritis score (Figures 7A, C).

### 3.4 Effect of NYT on blood cytokine levels

**FIGURE 8:**
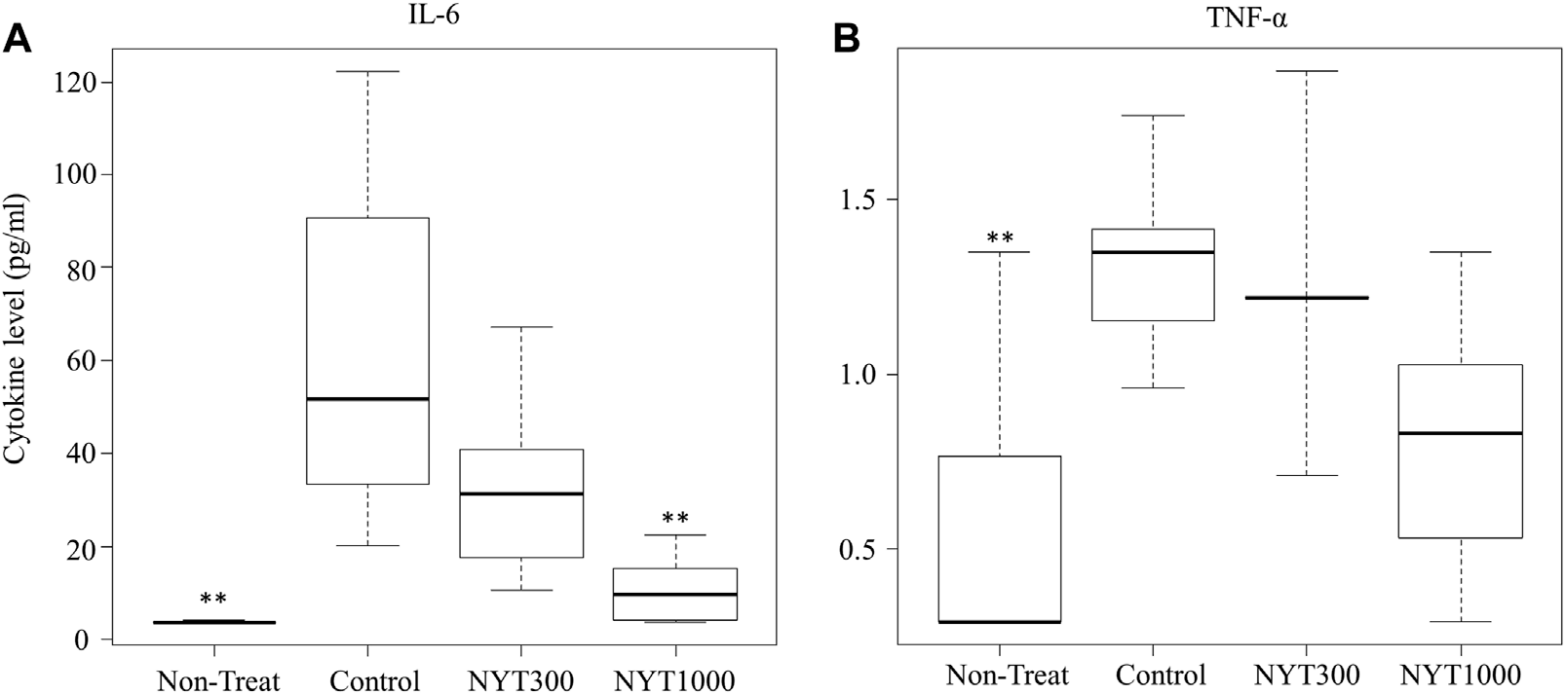
Effect of NYT on cytokine level in serum. **(A)** IL-6 level (Number of legs; No-Treatment *n* = 1, Control *n* = 7, NYT300 *n* = 5, NYT1000 *n* = 7). **(B)** TNF-α level (Number of legs; No-Treatment *n* = 7, Control *n* = 7, NYT300 *n* = 5, NYT1000 *n* = 7). The samples in each group that were below the detection threshold were excluded. Values are expressed as median [25th percentile, 75th percentile]. ∗∗*p* < 0.01 vs. control group by steel test.

Both IL-6 and TNF-α showed a significant increase in expression levels in the Control group compared to the No-Treatment group (Figure 8). We confirmed that the increase in blood IL-6 in the CIA model was reversed in the NYT1000 group (Figure 8A). No significant effectiveness in reversing IL-6 increase was found in the NYT300 group compared to the Control group. Analysis of blood TNF-α did not reveal a significant effect following NYT1000 administration (Figure 8B), but we have confirmed that TNF-α also tends to improve (*p* = 0.059).

## 4 Discussion

In this study, we found that *Ninjin’yoeito* was effective against the swelling and pain symptoms of rheumatoid arthritis and against associated muscle atrophy.

The arthritis score, which is an index of the degree of progression of rheumatoid arthritis, showed significant increases in the Control and NYT groups compared to the No-Treatment group 1 week after the second antigen administration. In addition, after the second experimental week, the increase in the arthritis score in the NYT1000 group was significantly reversed compared to the Control group. Therefore, we believe that NYT reversed the worsening of the swelling of rheumatoid arthritis.

The pain test we conducted to confirm changes in pain sensitivity showed that model mice became more susceptible to feeling pain following the onset of rheumatoid arthritis. Here, we found that the decrease in the pain threshold of mice treated with NYT were reversed in the fourth experimental week compared to the Control group. Our further analysis showed a negative correlation between the arthritis score and pain threshold. In addition, when focusing on the effect of NYT administration by arthritis pain score group, we confirmed that NYT administration increased the pain threshold. Therefore, we believe that the above two factors that suppression of arthritis and elevation of the pain threshold itself are involved in mediating the reversing effect of NYT administration on the decrease in pain threshold.

Our study of the skeletal muscle wet weight showed that administration of NYT1000 reversed the decrease in muscle wet weight. In other words, NYT administration reversed the sarcopenia that occurs due to rheumatoid arthritis. As for the decreased pain threshold, we believe that *Ninjin’yoeito* controls sarcopenia by reversing the increase in the arthritis score and suppressing muscle atrophy itself.

Our further analysis showed that the onset of muscle atrophy is dependent upon the arthritis score. Moreover, when we compared NYT administration vs non-administration within the same arthritis score group, we found a significant decrease in the muscle mass of the soleus and gastrocnemius muscles. Therefore, we believe the two above factors that suppression of arthritis and reduction of muscle weight loss are involved in mediating the suppression of muscle atrophy following NYT administration.

In this experiment, the soleus, plantaris, and gastrocnemius muscles were analyzed, and the effect of NYT was confirmed only in the soleus and gastrocnemius muscles. In our opinion, this is related to differences in the component muscle fiber types in the various muscles. Skeletal muscles can be broadly classified into type I fibers (slow and red muscles), which are capable of sustained activity, and type II fibers (fast and white muscles), which contract rapidly and strongly. Type II fibers are further divided into type IIA, which has a high aerobic metabolic capacity, and type IIB, which has a low aerobic metabolic capacity. It is generally believed that the soleus muscle is classified as type I fibers, the plantaris muscle as type IIB fibers, and the gastrocnemius muscle as type IIA fibers. Therefore, the present results suggest that NYT may affect skeletal muscle, especially type I and type IIA fibers.

Next, we focused on inflammatory cytokines. It has been reported that in rheumatoid arthritis, the blood levels of IL-6 and TNF-α increase (Ogata et al., 2012; Tanaka et al., 2014; Ogata et al., 2015). Thus, we measured the blood levels of inflammatory cytokines 4 weeks after the second antigen administration. The results of our analysis showed that NYT treatment significantly suppressed IL-6 expression and tended to suppress TNF-α expression.

IL-17 is another inflammatory cytokine that is related to rheumatoid arthritis. In the present study, we attempted to investigate the expression level of IL-17 in the blood, but it was not possible to detect (data not shown).

The onset of arthritis, pain, and muscle atrophy affected by NYT in this study are closely related to inflammatory cytokines.

Previous studies have reported a relationship between joint swelling and inflammatory cytokines. The synovial membranes located around the joints are involved in the joint swelling due to rheumatoid arthritis (Yukawa Rheumatology Clinic., 2017). In rheumatoid arthritis, T cells infiltrate the synovial membrane, produce inflammatory cytokines, and cause synovitis, which in turn causes joint swelling (Sato et al., 2006). Pain is divided into three categories depending on the cause: 1) Nociceptive pain; 2) Neuropathic pain; and 3) Psychogenic pain. The pain caused by rheumatoid arthritis is classified as nociceptive pain as inflammatory cytokines stimulate nociceptors in the peripheral nerves (Pain information site Totsu.jp., 2018-2021). In skeletal muscles, inflammatory cytokines are involved in multiple molecular pathways associated with protein metabolism, causing an imbalance between protein synthesis and breakdown, which in turn leads to a decrease in skeletal muscles (Goodman, 1991). Thus, the onset of arthritis, pain, and muscle atrophy in rheumatoid arthritis may result from the generation of inflammatory cytokines.

Recent studies have reported the therapeutic effect of ginsenosides, the main active ingredients of ginseng, on the CIA model (Meng et al., 2021). In a previous study, it was found that ginsenoside increased CD8 + T cell to down-regulate the immune response, reduced activated CD4 + T cell number and proinflammatory M1-macrophages resulted in the inhibition of the secretion of proinflammatory cytokine such as TNF-α and IL-6. Therefore, we believe that NYT acts on these T cells and macrophages to suppress the increase in inflammatory cytokines and reduce the symptoms of rheumatoid arthritis. How the suppression of proinflammatory cytokines by NYT leads to symptom control remains to be elucidated in many respects. We intend to work on clarifying these issues in the future.

One of the therapeutic drugs that are currently widely used for rheumatoid arthritis is a class known as anti-rheumatic drugs, which includes methotrexate (MTX). In recent years, biological drugs have also been found to be particularly effective against rheumatoid arthritis, and early treatment is said to cause remission. On the other hand, the fact that biological drugs are associated with the risk of infection and their high cost are viewed as limiting factors. In addition, their therapeutic effectiveness varies from person to person and they are ineffective on some people.

The merits of *kampo* include the fact that traditional drugs can compensate for the problems associated with Western medicine. *Kampo* have fewer adverse effects than Western medicine drugs and are less expensive. In actual clinical settings, *kampo* are prescribed for alleviating the adverse effects of Western medicines, and a previous study has reported that the combined use of Western medicine and *kampo* can help reduce costs (Ohno et al., 2013). Even today when there are many Western medicine drugs that are effective against rheumatoid arthritis, *kampo* may be one therapeutic option for the treatment of rheumatoid arthritis.

The present study showed that *ninjin’yoeito* promotes the normalization of the excessive expression of cytokines induced by rheumatoid arthritis and that it effectively relieves the symptoms of swelling and pain, which suggests that it may be useful in the suppression of secondary sarcopenia. In the future, we would like to further investigate the underlying mechanisms of action and the usefulness of *ninjin’yoeito* in treating autoimmune diseases such as rheumatoid arthritis and associated sarcopenia, which are health issues in a super-aging society.

## Data Availability

The original contributions presented in the study are included in the article/Supplementary Material, further inquiries can be directed to the corresponding author.
